# A Rare Case of Non-Hodgkin Lymphoma of the Tracheobronchial Tree

**DOI:** 10.7759/cureus.95979

**Published:** 2025-11-03

**Authors:** Nikhil P Jain, Narendra Hirani, Ajeet K Khilnani, Chandrark Oza, Karmi Patel

**Affiliations:** 1 Otolaryngology - Head and Neck Surgery, Gujarat Adani Institute of Medical Sciences, Bhuj, IND; 2 Pediatrics, Gujarat Adani Institute of Medical Sciences, Bhuj, IND; 3 Pathology, Gujarat Adani Institute of Medical Sciences, Bhuj, IND

**Keywords:** anaplastic variant diffuse large b-cell lymphoma, hodgkin lymphoma, horseshoe shaped nuclei, non hodgkin's lymphoma, tracheobronchial tree

## Abstract

Non-Hodgkin lymphoma (NHL) is a group of malignant neoplasms originating mainly from the lymph nodes. These tumors may result from chromosomal translocations, various toxins, infections, or chronic inflammation. NHL primarily involving the tracheobronchial tree is uncommon. Surgery, chemotherapy, and radiation therapy have been used alone or in combination for treatment. Here, we report a case of NHL of the tracheobronchial tree in a seven-year-old child, which was initially diagnosed as a foreign body in the left bronchus.

## Introduction

Malignant lymphomas are divided into Hodgkin's lymphomas (HL) and Non-Hodgkin lymphomas (NHL). NHL comprises approximately 5% of head and neck malignancies and displays a wide range of appearances compared to HL. Extra-nodal disease, with or without lymph node involvement, is more common among NHL patients. NHL primarily involving the trachea-bronchial tree accounts for less than 1% of all NHL patients [1]. Mesenchymal B and T cells, probably involved in immune surveillance of the upper airways, are the primary source of origin of tracheobronchial lymphomas. A localized tracheobronchial mass is a far less common pattern compared to the diffuse distribution pattern [2]. Respiratory failure can occur when NHL involves the central airway [3], making treatment difficult and leading to a poorer prognosis. Bronchoscopy and biopsy are essential to differentiate these lesions from primary bronchogenic carcinoma [4]. Chemotherapy with or without radiotherapy is mandatory for all patients with disseminated disease and for those patients with primary tracheobronchial NHL with residual disease after resection or if resection is not feasible [5].

## Case presentation

**Figure 1:**
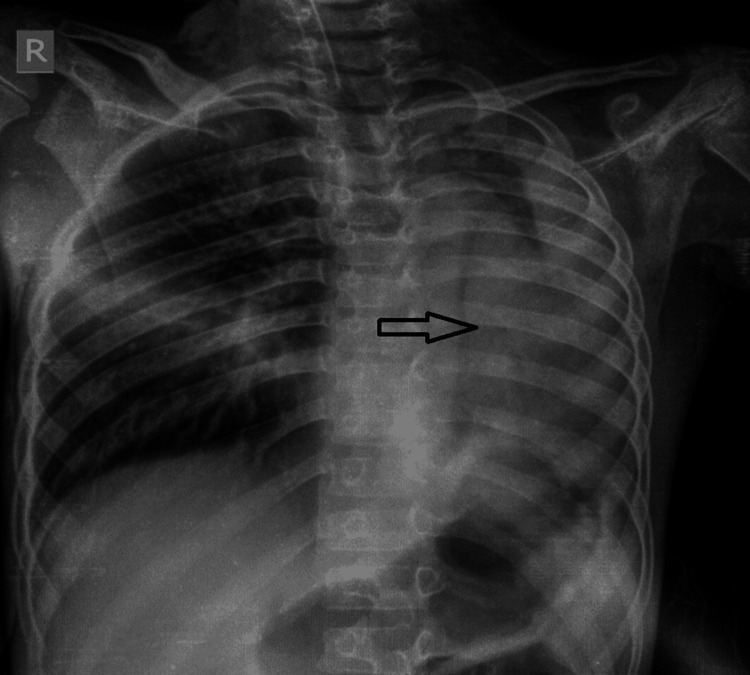
Chest X-ray showing a mediastinal shift toward the left side (black arrow) with volume loss due to left lung collapse

A seven-year-old male patient presented to the Pediatrics Department of a tertiary care hospital in western Gujarat with complaints of breathing difficulty and cough for the past four days. The patient's relative reported significant weight loss in the last two months and intermittent mild-grade fever episodes. Upon admission, the patient exhibited altered sensorium and tachypnea. He was placed on oxygen support via a non-rebreather mask. The patient had normal development for his age, was a school-going child, and had no significant past, personal, or family history. There was no lymph node enlargement in any other part of the body. A chest X-ray revealed homogeneously increased opacity of the left hemithorax with a mediastinal shift toward the left side (Figure 1).

**Figure 2:**
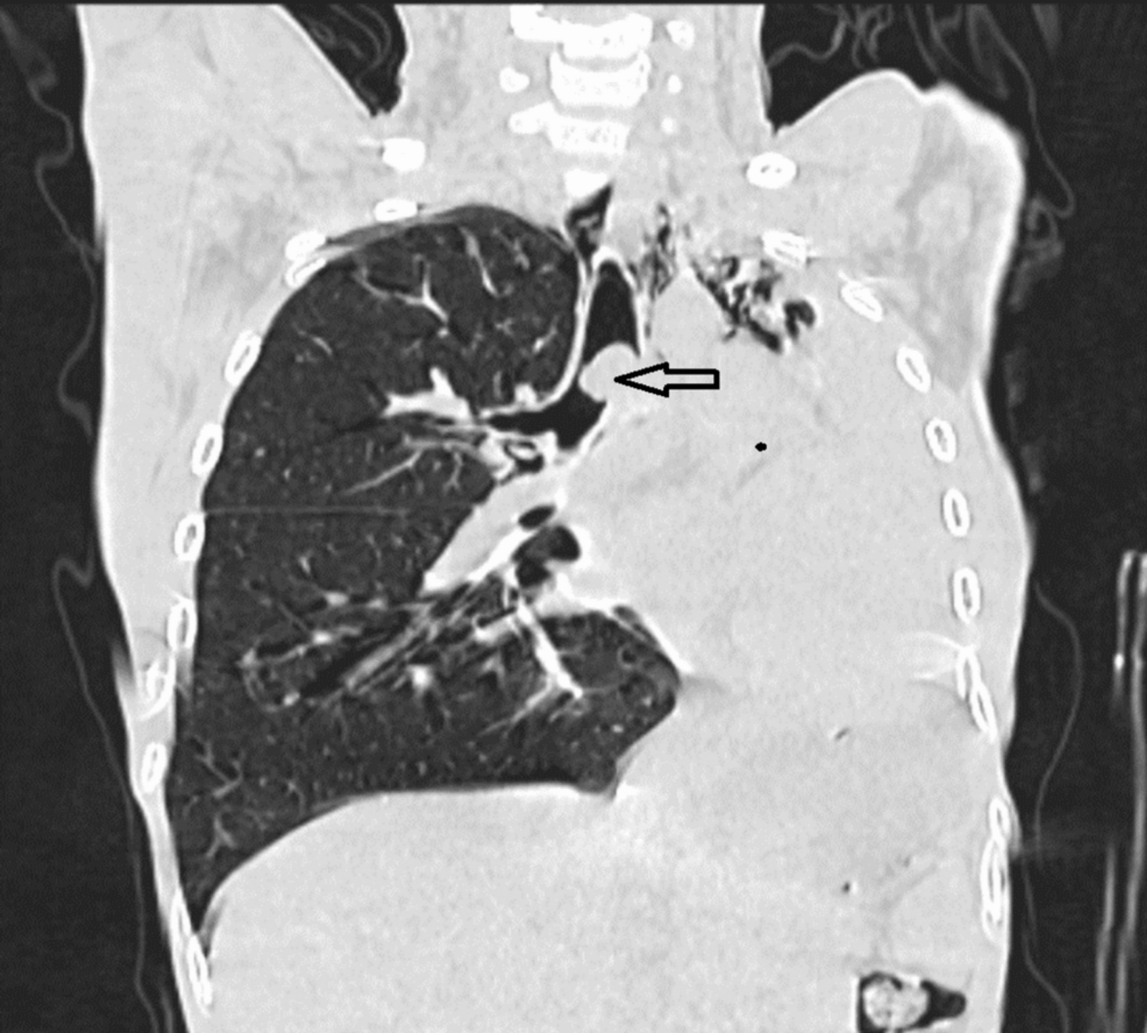
CECT of thorax showing a well-defined soft tissue density at the level of the tracheal bifurcation (black arrow) with lung collapse on the left side and compensatory hypertrophy on the right side CECT: contrast-enhanced computed tomography

These findings indicate volume loss due to the collapse of the left lung. Contrast-enhanced computed tomography (CECT) of the neck (Figure 2) and thorax showed a well-defined soft tissue density at the level of the tracheal bifurcation, causing moderate to severe narrowing of the trachea at the carinal level and complete occlusion of the left main stem bronchus. There was complete collapse-consolidation of the left upper and lower lobes with mild to moderate volume loss. Additionally, there was a shift of the trachea and mediastinum toward the left side, with compensatory hyperinflation of the right upper and lower lobes and transmediastinal herniation of the right lung toward the left side. These findings suggest the possibility of a foreign body or neoplastic etiology.

**Figure 3:**
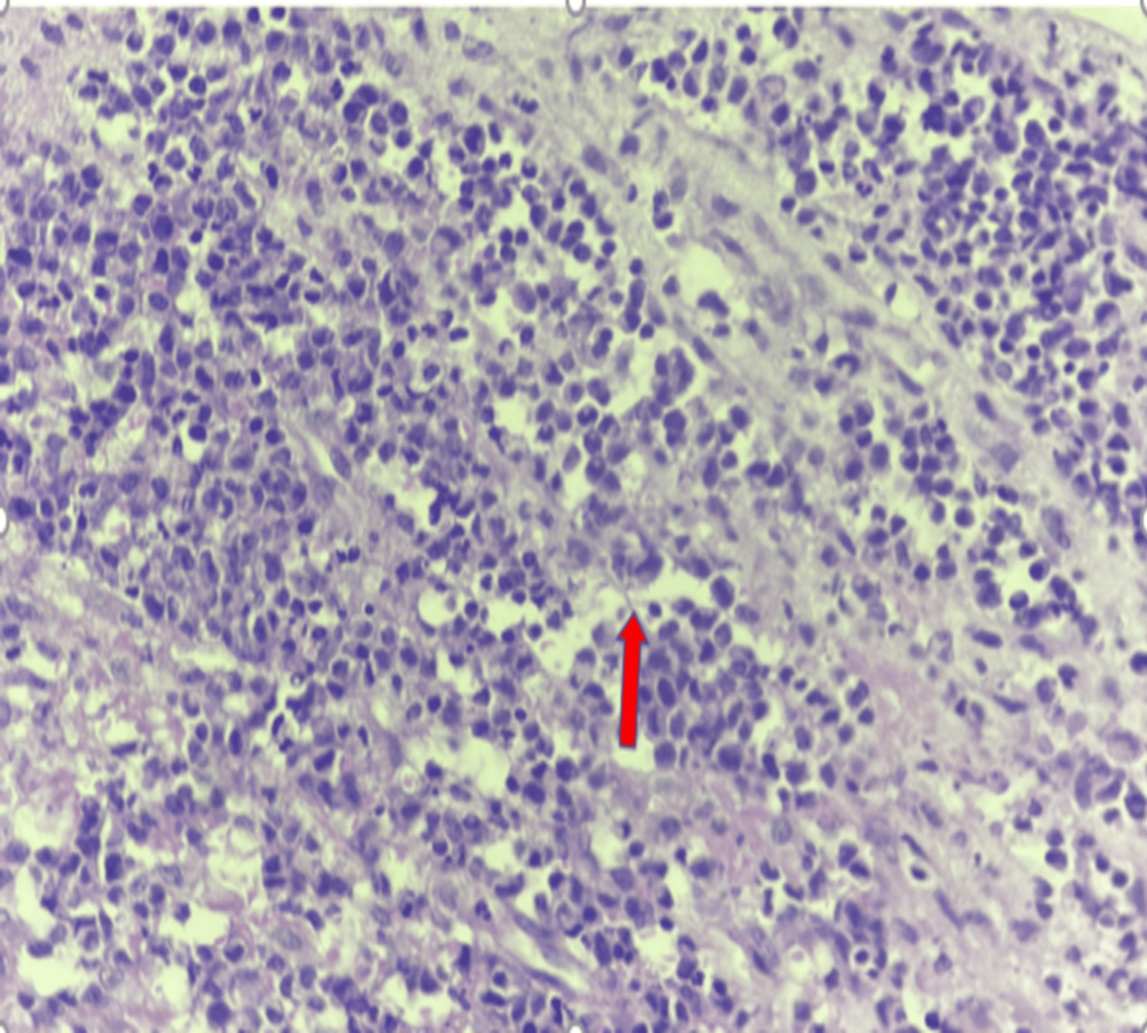
Red arrow showing the hallmark feature: horseshoe-shaped nuclei (H & E, 40x)

Bronchoscopy was planned. During the procedure, a soft tissue mass was visualized in the left main bronchus near the carina, which was identified as an obstructing mass rather than a foreign body. The mass was removed and sent for histopathological examination. Upon gross examination, multiple tissue fragments were observed, with a grayish-white color. The largest fragment measured 1.5 x 1 x 0.5 cm, while the remaining fragments measured 1.5 x 0.6 x 0.3 cm. Microscopic examination (Figure 3) revealed fragments of fibrocollagenous tissue infiltrated by tumor cells arranged in a diffuse pattern with a high nuclear-to-cytoplasmic (N:C) ratio. The tumor cells exhibited marked nuclear pleomorphism, prominent one to two nucleoli, a cleaved nucleus, open chromatin, and scant to moderate cytoplasm. Additionally, 5 out of 10 High-Power Field (HPF) mitotic figures were noted, along with a few atypical mitoses. Tingible body macrophages, areas of hemorrhage, and necrosis were also present.

Features were suggestive of a lymphoproliferative lesion, more in favor of NHL. Immunohistochemistry (IHC) was done for confirmation and further specification. IHC was diffuse positive for CD30 and CD246, focal positive for EMA, CD2, CD8, CD3, and high KI67. Markers like CD20, TDT, CD34, CK, and CD4 were negative. The final diagnosis was anaplastic lymphoma kinase (ALK) positive lymphoma involving the left main stem bronchus. The patient's relatives were counseled for tumor-specific chemotherapy. However, as of the last follow-up, the patient had not started chemotherapy.

## Discussion

The most common tracheobronchial tumor is squamous cell carcinoma, followed by adenoid cystic carcinomas [6]. Of all cases of tracheal tumors, 0.23% are primary tracheobronchial NHL, making it a rare entity. Primary tracheobronchial NHL affects only 3.6% of NHL patients with extra-nodal disease. Anaplastic large cell lymphoma comprises 2-7% of NHLs [4]. The first case of tracheobronchial NHL was described in 1955 by Dawe et al. [7]. The average age of presentation of primary tracheobronchial tumors is 44 years, with a range of 4-81 years [8]. Patients often present late, as the tumor is usually asymptomatic until 50% to 75% of the luminal diameter is occluded. Exertional dyspnea can occur when the tracheal lumen is narrowed to less than 7 mm. The common presenting features are dyspnea, cough, and wheezing, and are non-specific [9]. Dyspnea could be a clue to the severity of central airway obstruction. CECT and bronchoscopy are diagnostic. Other respiratory illnesses, like asthma and chronic obstructive pulmonary disease, which cause similar symptoms, can be differentiated by pulmonary function tests with flow volume curves at the initial presentation. Chemotherapy with the cyclophosphamide, hydroxydaunorubicin, oncovin, and prednisolone (CHOP) regimen is the first-line treatment, used in both ALK+ and ALK- types. The CHOEP (CHOP + etoposide) regimen is preferred in younger patients with ALK-positive lymphoma. Tumor resection or stent placement should be considered for large tumors [8]. Other treatment modalities include targeted therapy with brentuximab (an anti-CD30 antibody-drug conjugate), radiation therapy, and stem cell transplantation.

## Conclusions

Though rare, primary tracheobronchial NHL should be considered in the differential diagnosis of airway obstruction, as it can be fatal if untreated. Early diagnosis with CECT, biopsy, and bronchoscopic resection followed by chemotherapy helps the patient alleviate the airway obstruction. A multidisciplinary approach involving an otorhinolaryngologist, pediatrician, pathologist, and oncologist is crucial for the management of primary tracheobronchial NHL.
